# *rac*-(*E*,*E*)-*N*,*N*′-Bis(2-chloro­benzyl­idene)cyclo­hexane-1,2-di­amine

**DOI:** 10.1107/S1600536813014554

**Published:** 2013-06-12

**Authors:** Ismail Warad, Mousa Al-Noaimi, Salim F. Haddad, Yasmin Al-Demeri, Belkheir Hammouti

**Affiliations:** a Department of Chemistry, An-Najah National University, Nablus, State of Palestine; b Department of Chemistry, Hashemite University, Zarqa 13115, Jordan; c Department of Chemistry, University of Jordan, Amman 11942, Jordan; d LCAE-URAC18, Faculté des Sciences, Université Mohammed Ier, Oujda 60000, Morocco

## Abstract

In the title racemic Schiff base ligand, C_20_H_20_Cl_2_N_2_, which was prepared by the condensation of 2-chloro­benzaldehyde and cyclo­hexane-1,2-di­amine, the cyclo­hexane ring adopts a chair conformation and the dihedral angle between the aromatic rings of the 2-chloro­phenyl substituent groups is 62.52 (8)°. In the structure, there are two short intra­molecular methine C—H⋯Cl inter­actions [C⋯Cl = 3.066 (2) and 3.076 (3) Å], and in the crystal there are also weak inter­molecular aromatic C—H⋯Cl [3.464 (3), 3.553 (3) and 3.600 (3) Å] and Cl⋯Cl [3.557 (3) and 3.891 (3) Å] contacts.

## Related literature

For the crystal structures of some Schiff bases derived from cyclo­hexane-1,2-di­amine, see: Arvinnezhad *et al.* (2012 ▶); Fan *et al.* (2011 ▶); Saleh Salga *et al.* (2010 ▶). For applications of chiral Schiff base ligands, see: Da Silva *et al.* (2011 ▶); Dhar & Taploo (1982 ▶); Przybylski *et al.* (2009 ▶); Gupta & Sutar (2008 ▶). For the synthesis of the title compound, see: Larrow & Jacobsen (1998 ▶).
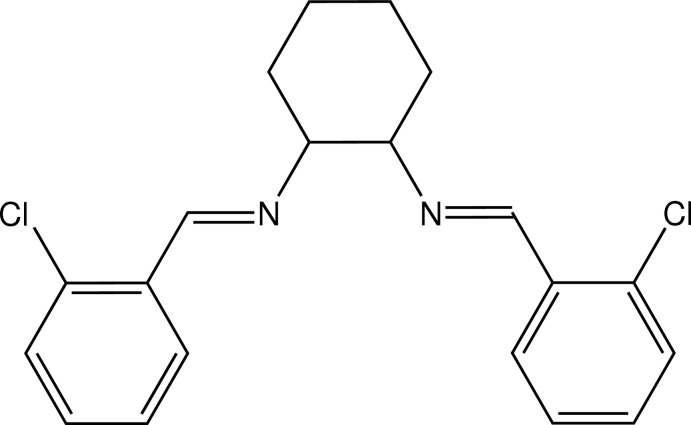


## Experimental

### 

#### Crystal data

C_20_H_20_Cl_2_N_2_
*M* *_r_* = 359.28
Monoclinic, 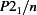

*a* = 5.9029 (5) Å
*b* = 19.5613 (13) Å
*c* = 16.1662 (11) Å
β = 93.493 (7)°
*V* = 1863.2 (2) Å^3^
*Z* = 4
Mo *K*α radiation
μ = 0.35 mm^−1^
*T* = 293 K
0.30 × 0.20 × 0.15 mm

#### Data collection

Agilent Xcalibur Eos diffractometer
Absorption correction: multi-scan (*CrysAlis PRO*; Agilent, 2011 ▶) *T*_min_ = 0.902, *T*_max_ = 0.949
7483 measured reflections
3273 independent reflections
2252 reflections with *I* > 2σ(*I*)
*R*_int_ = 0.030

#### Refinement

*R*[*F* ^2^ > 2σ(*F* ^2^)] = 0.046
*wR*(*F* ^2^) = 0.106
*S* = 1.02
3273 reflections
217 parameters
H-atom parameters constrained
Δρ_max_ = 0.28 e Å^−3^
Δρ_min_ = −0.20 e Å^−3^

### 

Data collection: *CrysAlis PRO* (Agilent, 2011 ▶); cell refinement: *CrysAlis PRO*; data reduction: *CrysAlis PRO*; program(s) used to solve structure: *SHELXS97* (Sheldrick, 2008 ▶); program(s) used to refine structure: *SHELXL97* (Sheldrick, 2008 ▶); molecular graphics: *ORTEPIII* (Burnett & Johnson, 1996 ▶); software used to prepare material for publication: *SHELXL97*.

## Supplementary Material

Crystal structure: contains datablock(s) I, global. DOI: 10.1107/S1600536813014554/zs2261sup1.cif

Structure factors: contains datablock(s) I. DOI: 10.1107/S1600536813014554/zs2261Isup2.hkl

Supplementary material file. DOI: 10.1107/S1600536813014554/zs2261Isup3.cml

Additional supplementary materials:  crystallographic information; 3D view; checkCIF report

## Figures and Tables

**Fig. 1.**
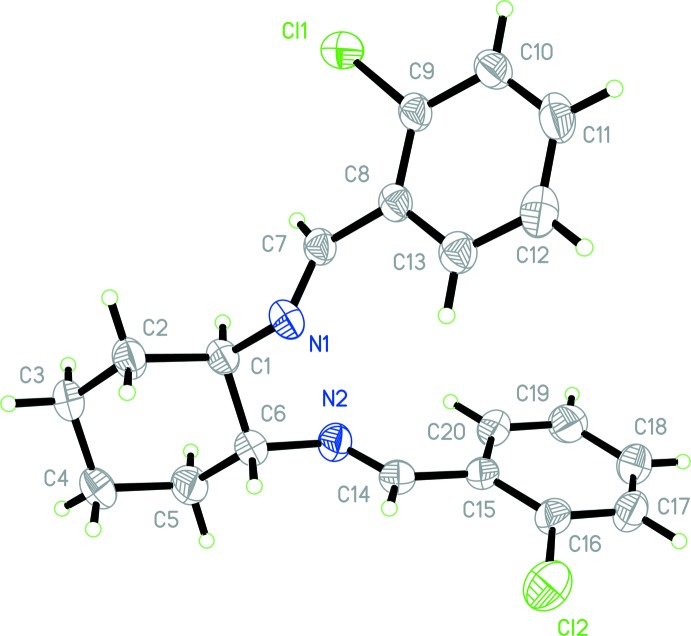
Molecular conformation and atom-numbering scheme for the title compound. Displacement ellipsoids are drawn at the 30% probability level. H atoms are represented as small spheres of arbitrary radii.

**Fig. 2.**
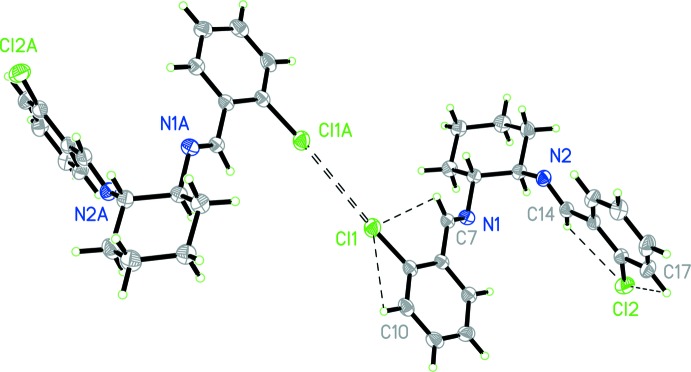
Molecular conformation showing intramolecular C7—H···Cl1, C10—H···Cl1, C14—H···Cl2 and C17—H···Cl2 contacts, as well as a short intermolecular Cl1···Cl1A contact. For symmetry code (A): -x + 1, -y, -z.

## supplementary crystallographic information

### Comment

The chelating chiral Schiff bases are significant compounds in chemistry so that several reviews have been published on these substances (Gupta & Sutar, 2008; Da Silva *et al.*, 2011; Przybylski *et al.*, 2009). Because of their stereochemical features, as well as their industrial properties (Dhar & Taploo, 1982) and potent biological activities (Da Silva *et al.*, 2011; Przybylski *et al.*, 2009), they are very attractive synthetic targets. Furthermore, it should be stressed that these useful and recyclable chemicals have been widely used in various enantioselective reactions, such as cyclopropanation, aziridination, epoxidation or the Diels–Alder reaction, and as ligands or catalysts.

The title Schiff base, C_20_H_20_Cl_2_N_2_, was prepared by condensation of commercially available 2-chlorobenzaldehyde and (1*R*,2*R*)-diaminocyclohexane and the structure is reported herein. However, this compound is racemic, in which the cyclohexane ring adopts the expected chair conformation, with a dihedral angle of 62.52 (8)° between the aromatic rings of the two 2-chlorophenyl substituent groups (Fig. 1). The structure of the chiral isomeric (1*R*,2*R*) 4-chlorophenyl analogue has been reported (Arvinnezhad *et al.*, 2012). In the title compound, the conformation is stabilized by intramolecular C7—H···Cl1 and C14—H··· Cl2 interactions [3.066 (2) and 3.076 (3) Å, respectively] (Table 1). In the crystal there are weak intermolecular methine C—H···Cl interactions [C10—H···Cl1 [3.600 (3) Å] (-*x* + 2, -*y*, -*z*), C11—H···Cl1 [3.553 (3) Å] (*x* - 1, *y*, *z*) and C20—H···Cl2 [3.464 (3) Å] (1 + *x* + 1, *y*, *z*). Also present in the crystal are Cl···Cl contacts [Cl1···Cl1, 3.557 (3) Å (-*x* + 1, -*y*, -*z*)] and 3.891 (3) Å (-*x* + 2, -*y*, -*z*) (Fig. 2).

### Experimental

(*R*,*R*)-1,2-Diaminocyclohexane (1 g, 8.9 mmol) was dissolved in EtOH (10 ml) and the mixture was stirred and heated gently (50 °C) for 10 min, after which a solution of 2-chlorobenzaldehyde (2.6 g, 18 mmol, 2 equivalents) in EtOH (5 ml) was added dropwise. The stirred reaction mixture was refluxed for a period of 4 h, with the reaction progress monitored by thin-layer chromatography. Upon completion of the reaction, the mixture was cooled to room temperature and the solid obtained was filtered off, washed with cold water and crystallized from ethanol (95%), with a 85% yield.

### Refinement

H atoms were positioned geometrically with C—H = 0.93 Å (aromatic), 0.97 Å (methylene) or 0.98 Å (methine) and allowed to ride in the refinement, with *U*_iso_(H) = 1.2*U*_eq_(C). The largest difference peak and hole are 0.276 and -0.204 e Å^-3^.

### Crystal data

**Table:**

C_20_H_20_Cl_2_N_2_	*F*(000) = 752
*M**_r_* = 359.28	*D*_x_ = 1.281 Mg m^−^^3^
Monoclinic, *P*2_1_/*n*	Mo *K*α radiation, λ = 0.71073 Å
Hall symbol: -P 2yn	Cell parameters from 2442 reflections
*a* = 5.9029 (5) Å	θ = 3.1–29.1°
*b* = 19.5613 (13) Å	µ = 0.35 mm^−^^1^
*c* = 16.1662 (11) Å	*T* = 293 K
β = 93.493 (7)°	Block, colourless
*V* = 1863.2 (2) Å^3^	0.30 × 0.20 × 0.15 mm
*Z* = 4	

### Data collection

**Table:**

Agilent Xcalibur Eos diffractometer	3273 independent reflections
Radiation source: Enhance (Mo) X-ray Source	2252 reflections with *I* > 2σ(*I*)
Graphite monochromator	*R*_int_ = 0.030
Detector resolution: 16.0534 pixels mm^-1^	θ_max_ = 25.0°, θ_min_ = 3.3°
ω scans	*h* = −7→6
Absorption correction: multi-scan (*CrysAlis PRO*; Agilent, 2011)	*k* = −23→18
*T*_min_ = 0.902, *T*_max_ = 0.949	*l* = −19→17
7483 measured reflections	

### Refinement

**Table:**

Refinement on *F*^2^	Primary atom site location: structure-invariant direct methods
Least-squares matrix: full	Secondary atom site location: difference Fourier map
*R*[*F*^2^ > 2σ(*F*^2^)] = 0.046	Hydrogen site location: inferred from neighbouring sites
*wR*(*F*^2^) = 0.106	H-atom parameters constrained
*S* = 1.02	*w* = 1/[σ^2^(*F*_o_^2^) + (0.0349*P*)^2^ + 0.4202*P*] where *P* = (*F*_o_^2^ + 2*F*_c_^2^)/3
3273 reflections	(Δ/σ)_max_ < 0.001
217 parameters	Δρ_max_ = 0.28 e Å^−^^3^
0 restraints	Δρ_min_ = −0.20 e Å^−^^3^

### Special details

**Table:**

Geometry. All e.s.d.'s (except the e.s.d. in the dihedral angle between two l.s. planes) are estimated using the full covariance matrix. The cell e.s.d.'s are taken into account individually in the estimation of e.s.d.'s in distances, angles and torsion angles; correlations between e.s.d.'s in cell parameters are only used when they are defined by crystal symmetry. An approximate (isotropic) treatment of cell e.s.d.'s is used for estimating e.s.d.'s involving l.s. planes.
Refinement. Refinement of *F*^2^ against ALL reflections. The weighted *R*-factor *wR* and goodness of fit *S* are based on *F*^2^, conventional *R*-factors *R* are based on *F*, with *F* set to zero for negative *F*^2^. The threshold expression of *F*^2^ > σ(*F*^2^) is used only for calculating *R*-factors(gt) *etc*. and is not relevant to the choice of reflections for refinement. *R*-factors based on *F*^2^ are statistically about twice as large as those based on *F*, and *R*-factors based on ALL data will be even larger.

### Fractional atomic coordinates and isotropic or equivalent isotropic displacement parameters (Å^2^)

**Table:**

	*x*	*y*	*z*	*U*_iso_*/*U*_eq_	
Cl1	0.73684 (13)	0.05324 (3)	0.02770 (4)	0.0762 (2)	
Cl2	1.12436 (13)	0.46542 (4)	0.17646 (5)	0.0914 (3)	
N1	0.7836 (3)	0.26396 (10)	−0.03727 (11)	0.0555 (5)	
C8	0.9564 (4)	0.17516 (12)	0.04725 (12)	0.0489 (6)	
C1	0.5739 (4)	0.29405 (11)	−0.07382 (12)	0.0520 (6)	
H1B	0.4442	0.2674	−0.0567	0.062*	
C6	0.5559 (4)	0.36693 (12)	−0.04157 (13)	0.0583 (7)	
H6A	0.6885	0.3932	−0.0568	0.070*	
N2	0.5510 (4)	0.36480 (10)	0.04896 (11)	0.0613 (6)	
C14	0.7170 (5)	0.39062 (12)	0.09007 (14)	0.0586 (6)	
H14A	0.8318	0.4110	0.0617	0.070*	
C7	0.7660 (4)	0.20790 (12)	−0.00033 (12)	0.0492 (6)	
H7A	0.6258	0.1861	−0.0032	0.059*	
C13	1.1395 (4)	0.21396 (13)	0.07903 (13)	0.0587 (6)	
H13A	1.1465	0.2602	0.0661	0.070*	
C15	0.7373 (4)	0.38992 (12)	0.18143 (14)	0.0550 (6)	
C10	1.1237 (5)	0.07662 (14)	0.11857 (14)	0.0673 (8)	
H10A	1.1179	0.0304	0.1317	0.081*	
C9	0.9546 (4)	0.10611 (12)	0.06799 (12)	0.0540 (6)	
C16	0.9153 (4)	0.42153 (12)	0.22641 (15)	0.0615 (7)	
C20	0.5769 (5)	0.35715 (13)	0.22604 (15)	0.0669 (7)	
H20A	0.4546	0.3359	0.1976	0.080*	
C11	1.3010 (5)	0.11644 (17)	0.14930 (15)	0.0777 (9)	
H11A	1.4156	0.0972	0.1838	0.093*	
C17	0.9323 (5)	0.42009 (15)	0.31214 (17)	0.0756 (8)	
H17A	1.0535	0.4415	0.3411	0.091*	
C2	0.5740 (4)	0.29389 (13)	−0.16818 (13)	0.0634 (7)	
H2B	0.7073	0.3178	−0.1853	0.076*	
H2C	0.5804	0.2472	−0.1880	0.076*	
C3	0.3615 (5)	0.32848 (13)	−0.20597 (14)	0.0679 (7)	
H3A	0.3679	0.3298	−0.2658	0.081*	
H3B	0.2292	0.3020	−0.1931	0.081*	
C5	0.3413 (5)	0.40078 (14)	−0.07892 (15)	0.0783 (9)	
H5A	0.3340	0.4476	−0.0594	0.094*	
H5B	0.2093	0.3766	−0.0610	0.094*	
C12	1.3100 (4)	0.18501 (17)	0.12915 (15)	0.0725 (8)	
H12A	1.4315	0.2116	0.1495	0.087*	
C19	0.5930 (5)	0.35511 (14)	0.31149 (17)	0.0783 (8)	
H19A	0.4841	0.3322	0.3401	0.094*	
C4	0.3382 (5)	0.40022 (14)	−0.17353 (16)	0.0825 (9)	
H4A	0.1970	0.4199	−0.1962	0.099*	
H4B	0.4620	0.4281	−0.1915	0.099*	
C18	0.7716 (6)	0.38728 (15)	0.35396 (17)	0.0819 (9)	
H18A	0.7825	0.3866	0.4116	0.098*	

### Atomic displacement parameters (Å^2^)

**Table:**

	*U*^11^	*U*^22^	*U*^33^	*U*^12^	*U*^13^	*U*^23^
Cl1	0.0871 (5)	0.0566 (4)	0.0835 (5)	0.0021 (4)	−0.0058 (4)	0.0000 (3)
Cl2	0.0686 (5)	0.1026 (6)	0.1027 (6)	−0.0136 (4)	0.0018 (4)	−0.0098 (4)
N1	0.0581 (13)	0.0600 (13)	0.0482 (11)	0.0065 (10)	0.0025 (10)	0.0112 (9)
C8	0.0547 (15)	0.0576 (15)	0.0350 (11)	0.0102 (12)	0.0076 (10)	−0.0003 (10)
C1	0.0560 (15)	0.0538 (14)	0.0457 (12)	0.0028 (12)	0.0003 (11)	0.0077 (10)
C6	0.0690 (17)	0.0549 (15)	0.0499 (13)	0.0001 (13)	−0.0050 (12)	0.0039 (11)
N2	0.0686 (14)	0.0649 (13)	0.0497 (11)	0.0024 (11)	−0.0019 (10)	−0.0047 (9)
C14	0.0677 (17)	0.0493 (14)	0.0583 (15)	0.0044 (13)	0.0002 (13)	−0.0018 (11)
C7	0.0542 (14)	0.0543 (14)	0.0393 (11)	0.0055 (12)	0.0034 (10)	−0.0002 (11)
C13	0.0649 (17)	0.0636 (16)	0.0478 (13)	0.0032 (13)	0.0052 (12)	−0.0007 (11)
C15	0.0638 (16)	0.0454 (14)	0.0547 (14)	0.0065 (12)	−0.0065 (13)	−0.0056 (11)
C10	0.087 (2)	0.0641 (17)	0.0496 (14)	0.0270 (16)	−0.0021 (14)	−0.0012 (12)
C9	0.0653 (16)	0.0589 (15)	0.0376 (12)	0.0145 (13)	0.0024 (11)	−0.0025 (10)
C16	0.0647 (17)	0.0533 (15)	0.0656 (16)	0.0062 (13)	−0.0041 (13)	−0.0067 (12)
C20	0.0782 (19)	0.0636 (17)	0.0576 (16)	−0.0079 (15)	−0.0060 (14)	−0.0053 (12)
C11	0.081 (2)	0.097 (2)	0.0536 (15)	0.0358 (18)	−0.0137 (15)	−0.0084 (15)
C17	0.081 (2)	0.076 (2)	0.0680 (18)	0.0003 (17)	−0.0157 (16)	−0.0181 (15)
C2	0.0752 (18)	0.0686 (17)	0.0461 (13)	0.0056 (14)	0.0021 (13)	0.0066 (12)
C3	0.0817 (19)	0.0721 (18)	0.0481 (14)	0.0024 (15)	−0.0103 (13)	0.0079 (12)
C5	0.096 (2)	0.0648 (17)	0.0712 (18)	0.0260 (16)	−0.0146 (16)	−0.0042 (14)
C12	0.0621 (17)	0.098 (2)	0.0570 (16)	0.0101 (17)	−0.0040 (14)	−0.0143 (15)
C19	0.095 (2)	0.0727 (19)	0.0674 (18)	−0.0071 (17)	0.0074 (16)	0.0016 (14)
C4	0.100 (2)	0.0716 (19)	0.0717 (18)	0.0166 (17)	−0.0243 (17)	0.0146 (14)
C18	0.111 (3)	0.077 (2)	0.0552 (16)	0.0005 (19)	−0.0075 (18)	−0.0097 (14)

### Geometric parameters (Å, º)

**Table:**

Cl1—C9	1.745 (2)	C10—H10A	0.9300
Cl2—C16	1.742 (3)	C16—C17	1.384 (3)
N1—C7	1.256 (3)	C20—C19	1.379 (3)
N1—C1	1.462 (3)	C20—H20A	0.9300
C8—C9	1.392 (3)	C11—C12	1.382 (4)
C8—C13	1.393 (3)	C11—H11A	0.9300
C8—C7	1.469 (3)	C17—C18	1.359 (4)
C1—C6	1.524 (3)	C17—H17A	0.9300
C1—C2	1.525 (3)	C2—C3	1.520 (3)
C1—H1B	0.9800	C2—H2B	0.9700
C6—N2	1.466 (3)	C2—H2C	0.9700
C6—C5	1.522 (3)	C3—C4	1.507 (4)
C6—H6A	0.9800	C3—H3A	0.9700
N2—C14	1.256 (3)	C3—H3B	0.9700
C14—C15	1.475 (3)	C5—C4	1.529 (3)
C14—H14A	0.9300	C5—H5A	0.9700
C7—H7A	0.9300	C5—H5B	0.9700
C13—C12	1.375 (3)	C12—H12A	0.9300
C13—H13A	0.9300	C19—C18	1.375 (4)
C15—C20	1.383 (3)	C19—H19A	0.9300
C15—C16	1.386 (3)	C4—H4A	0.9700
C10—C11	1.373 (4)	C4—H4B	0.9700
C10—C9	1.378 (3)	C18—H18A	0.9300
			
C7—N1—C1	116.8 (2)	C15—C20—H20A	119.1
C9—C8—C13	117.2 (2)	C10—C11—C12	120.3 (2)
C9—C8—C7	122.2 (2)	C10—C11—H11A	119.9
C13—C8—C7	120.5 (2)	C12—C11—H11A	119.9
N1—C1—C6	108.26 (18)	C18—C17—C16	119.8 (3)
N1—C1—C2	110.58 (19)	C18—C17—H17A	120.1
C6—C1—C2	110.38 (18)	C16—C17—H17A	120.1
N1—C1—H1B	109.2	C3—C2—C1	110.5 (2)
C6—C1—H1B	109.2	C3—C2—H2B	109.5
C2—C1—H1B	109.2	C1—C2—H2B	109.5
N2—C6—C5	110.0 (2)	C3—C2—H2C	109.5
N2—C6—C1	108.72 (18)	C1—C2—H2C	109.5
C5—C6—C1	110.19 (19)	H2B—C2—H2C	108.1
N2—C6—H6A	109.3	C4—C3—C2	111.4 (2)
C5—C6—H6A	109.3	C4—C3—H3A	109.3
C1—C6—H6A	109.3	C2—C3—H3A	109.3
C14—N2—C6	117.0 (2)	C4—C3—H3B	109.3
N2—C14—C15	122.6 (3)	C2—C3—H3B	109.3
N2—C14—H14A	118.7	H3A—C3—H3B	108.0
C15—C14—H14A	118.7	C6—C5—C4	110.7 (2)
N1—C7—C8	123.1 (2)	C6—C5—H5A	109.5
N1—C7—H7A	118.4	C4—C5—H5A	109.5
C8—C7—H7A	118.4	C6—C5—H5B	109.5
C12—C13—C8	121.1 (3)	C4—C5—H5B	109.5
C12—C13—H13A	119.5	H5A—C5—H5B	108.1
C8—C13—H13A	119.5	C13—C12—C11	120.1 (3)
C20—C15—C16	117.0 (2)	C13—C12—H12A	119.9
C20—C15—C14	120.7 (2)	C11—C12—H12A	119.9
C16—C15—C14	122.3 (3)	C18—C19—C20	119.4 (3)
C11—C10—C9	119.1 (3)	C18—C19—H19A	120.3
C11—C10—H10A	120.5	C20—C19—H19A	120.3
C9—C10—H10A	120.5	C3—C4—C5	111.0 (2)
C10—C9—C8	122.2 (2)	C3—C4—H4A	109.4
C10—C9—Cl1	117.6 (2)	C5—C4—H4A	109.4
C8—C9—Cl1	120.09 (17)	C3—C4—H4B	109.4
C17—C16—C15	121.5 (3)	C5—C4—H4B	109.4
C17—C16—Cl2	117.6 (2)	H4A—C4—H4B	108.0
C15—C16—Cl2	120.8 (2)	C17—C18—C19	120.3 (3)
C19—C20—C15	121.9 (2)	C17—C18—H18A	119.8
C19—C20—H20A	119.1	C19—C18—H18A	119.8
			
C7—N1—C1—C6	126.4 (2)	C20—C15—C16—C17	0.3 (4)
C7—N1—C1—C2	−112.5 (2)	C14—C15—C16—C17	−179.7 (2)
N1—C1—C6—N2	−60.1 (3)	C20—C15—C16—Cl2	−178.99 (19)
C2—C1—C6—N2	178.69 (19)	C14—C15—C16—Cl2	1.0 (3)
N1—C1—C6—C5	179.2 (2)	C16—C15—C20—C19	−0.7 (4)
C2—C1—C6—C5	58.1 (3)	C14—C15—C20—C19	179.3 (2)
C5—C6—N2—C14	−125.3 (2)	C9—C10—C11—C12	−0.5 (4)
C1—C6—N2—C14	114.0 (2)	C15—C16—C17—C18	−0.2 (4)
C6—N2—C14—C15	−178.0 (2)	Cl2—C16—C17—C18	179.1 (2)
C1—N1—C7—C8	−173.03 (19)	N1—C1—C2—C3	−177.1 (2)
C9—C8—C7—N1	−161.7 (2)	C6—C1—C2—C3	−57.3 (3)
C13—C8—C7—N1	23.4 (3)	C1—C2—C3—C4	56.3 (3)
C9—C8—C13—C12	−0.4 (3)	N2—C6—C5—C4	−177.2 (2)
C7—C8—C13—C12	174.8 (2)	C1—C6—C5—C4	−57.3 (3)
N2—C14—C15—C20	3.3 (4)	C8—C13—C12—C11	−0.4 (4)
N2—C14—C15—C16	−176.7 (2)	C10—C11—C12—C13	0.9 (4)
C11—C10—C9—C8	−0.4 (4)	C15—C20—C19—C18	0.9 (4)
C11—C10—C9—Cl1	178.1 (2)	C2—C3—C4—C5	−55.8 (3)
C13—C8—C9—C10	0.8 (3)	C6—C5—C4—C3	56.3 (3)
C7—C8—C9—C10	−174.3 (2)	C16—C17—C18—C19	0.5 (4)
C13—C8—C9—Cl1	−177.66 (17)	C20—C19—C18—C17	−0.8 (4)
C7—C8—C9—Cl1	7.3 (3)		

### Hydrogen-bond geometry (Å, º)

**Table:**

*D*—H···*A*	*D*—H	H···*A*	*D*···*A*	*D*—H···*A*
C7—H7*A*···Cl1	0.93	2.72	3.066 (2)	103
C14—H14*A*···Cl2	0.93	2.68	3.076 (3)	107
